# Zoonotic Transmission of *Blastocystis* Subtype 1 among People in Eastern Communities of Thailand: Organic Fertilizer from Pig Feces as a Potential Source

**DOI:** 10.1128/Spectrum.00362-21

**Published:** 2021-09-29

**Authors:** Toon Ruang-areerate, Phunlerd Piyaraj, Picha Suwannahitatorn, Panthita Ruang-areerate, Thunyapit Thita, Tawee Naaglor, Umaporn Witee, Boonsub Sakboonyarat, Saovanee Leelayoova, Mathirut Mungthin

**Affiliations:** a Department of Parasitology, Phramongkutklao College of Medicine, Bangkok, Thailand; b National Omics Center, National Science and Technology Development Agency (NSTDA), Pathum Thani, Thailand; c Drug Research Unit for Malaria, Faculty of Tropical Medicine, Mahidol University, Bangkok, Thailand; d Department of Military and Community Medicine, Phramongkutklao College of Medicine, Bangkok, Thailand; Pennsylvania State University

**Keywords:** *Blastocystis*, zoonotic transmission, prevalence, fertilizer, subtype, Thailand

## Abstract

*Blastocystis* sp., the most common intestinal protozoa, remains a public health problem among people in many countries, particularly in rural areas of developing countries. The infection usually reflects poor sanitation in communities by waterborne, zoonotic, and person-to-person transmission. Interestingly, at least 17 subtypes (STs) have been reported and are associated with a broad range of animal hosts, including humans. In this study, we reported potential evidence of zoonotic transmission of *Blastocystis* ST1 in rural communities of eastern Thailand where the overall prevalence of *Blastocystis* infection was 15.7%. Two major and three minor subtypes were found to be distributed unequally in this region. Of 5 STs, only ST1 was found to be associated with pig feces in an open farm system that produced organic fertilizer for agriculture uses in the community. This finding suggests that properly protective contact and standard production of organic fertilizer from pig feces by-products could be key factors for reducing the prevalence of *Blastocystis* infection and prevent *Blastocystis* reinfection among people in the community.

**IMPORTANCE** *Blastocystis* sp. remains a public health problem among people, particularly in rural areas of many developing countries. The infection usually reflects poor sanitation in communities by waterborne, zoonotic, and person-to-person transmission. In this study, we reported potential evidence of zoonotic transmission of *Blastocystis* subtype 1 (ST1) in rural communities of eastern Thailand. Two major and three minor subtypes were found to be unequally distributed in this region. Interestingly, only ST1 was found to be associated with pig feces in an open farm system that produced organic fertilizer for agriculture uses in the community. The finding makes significant contributions to genetic and molecular investigations of microbial topics of practical value and suggest that properly protective contact and standard production of organic fertilizer from pig feces by-products could be key factors for reducing the prevalence of *Blastocystis* infection and prevent *Blastocystis* reinfection among people in the community.

## INTRODUCTION

*Blastocystis* sp. is an anaerobic enteric parasite, a member of the *Stramenopiles* or *Heterokonta* branch of the *Eukarya* (1), that is phylogenetically distinct from other microbial species of *Eukarya* and, inhabits or infects the human intestinal tract. It comprises one of the most common intestinal parasites, has a worldwide distribution (2), infects approximately between 1 and 2 billion people, and is frequently found in rural areas in developing countries (3). In developing countries, the prevalence is usually high, for example, 14.8% in Lao People’s Democratic Republic (PDR) (4), 18.4% in Cambodia (5), 13.3% in Malaysia (6), and 6.6% to 37.4% in Thailand (7–10). Most infections were asymptomatic, whereas some patients presented acute or chronic gastrointestinal illness (11–13). Infection with *Blastocystis* sp. can cause unspecified gastrointestinal symptoms, including abdominal pain, flatulence, constipation, diarrhea, nausea, and vomiting (14–16). In addition to infecting humans, *Blastocystis* sp. are common gastrointestinal tract protozoa of a wide range of animal hosts, such as livestock, primates, reptiles, and insects (17–19). At least 17 subtypes (STs) have been characterized, and human populations have been associated with 10 STs, including ST1-9 and ST12 (20); however, the majority of human-associated STs (∼90%) are categorized in 1 of 4 STs, including ST1, ST2, ST3, and ST4 (19). Contrastingly, ST13 to ST17 were found in a variety of nonhuman hosts based on nearly full-length small subunit (SSU) gene sequences (20). Independent risk factors associated with *Blastocystis* infection were animal handling (16, 21, 22), infected individuals (9, 10), and contaminated water (9, 23–25). Regarding the association with waterborne transmission (23, 26) as well as common occurrence in rural areas of developing countries (27), the infection may reflect the poor sanitation of the communities. Understanding the intermediate factors causing the distribution of *Blastocystis* sp. in the community is a crucial key for preventing the transmission among people. However, they remain unclearly known.

In this study, potential sources of *Blastocystis* transmission were examined among people in agricultural communities of eastern Thailand. We found that the *Blastocystis* infection was associated with the open pig farm system. Genetic characterization demonstrated zoonotic transmission of the *Blastocystis* sp. ST1 among residents in an eastern community of Thailand where at least five subtypes were distributed in the community.

## RESULTS

### Prevalence and risk factors of *Blastocystis* infection.

In January 2018, 902 participants were enrolled in this study and 745 stool samples were collected with a response rate of 82.6%. The characteristics of the enrolled subjects and the prevalence of *Blastocystis* infection are shown in Table 1. Of the 745 participants, 334 (44.8%) were male and 411 (55.2%) were female. The overall prevalence of *Blastocystis* infection was 15.7% (117 of 745). The prevalence of *Blastocystis* infection significantly differed regarding age group (*P* < 0.01), occupation (*P* < 0.01), education (*P* = 0.03) and among participants residing in different villages (*P* = 0.01). No significant difference was found regarding sex. The populations or households were mostly farmers and those graduated at primary school level or below.

**TABLE 1:** Characteristics of the enrolled subjects and the prevalence of *Blastocystis* infection

Characteristic	No. of enrolled subjects (%)	No. of infected subjects (%)	*P* value
Sex			
Male	334 (44.8)	49 (41.9)	
Female	411 (55.2)	68 (58.1)	0.48
Age (yr)			
<20	108 (14.5)	11 (9.4)	
20–29	18 (2.4)	3 (2.6)	
30–39	66 (8.9)	6 (5.1)	
40–49	161 (21.6)	40 (34.2)	
50–59	202 (27.1)	32 (27.4)	
>60	190 (25.5)	25 (21.4)	<0.01
Occupation			
Agriculture	426 (57.2)	61 (52.1)	
Animal career	102 (13.7)	40 (34.2)	
Other	217 (29.1)	16 (13.7)	<0.01
Education			
Primary school or below	559 (75.0)	97 (82.9)	
Middle school or above	186 (25.0)	20 (17.1)	0.03
Village			
No.1 (Moo 11)	85 (11.4)	12 (10.3)	
No.2 (Moo 11)	69 (9.3)	10 (8.5)	
No.3 (Moo 11)	77 (10.3)	10 (8.5)	
No.4 (Moo 11)	52 (7.0)	6 (5.1)	
No.5 (Moo 11)	64 (8.6)	10 (8.5)	
No.6 (Moo 11)	78 (10.5)	24 (20.5)	
No.7 (Moo 18)	98 (13.2)	22 (18.8)	
No.8 (Moo 18)	56 (7.5)	6 (5.1)	
No.9 (Moo 18)	59 (7.9)	5 (4.3)	
No.10 (Moo 18)	48 (6.4)	5 (4.3)	
No.11 (Moo 18)	59 (7.9)	7 (6.0)	0.01

A univariate analysis of the risk association for *Blastocystis* infection in this community showed that those at middle age from 40 to 49 years old (odds ratio [OR], 2.9; 95% confidence interval [CI], 1.4 to 6.0), living in Non Sa Ard or Tun Geang Village (OR, 2.5; 95% CI, 1.6 to 3.8), working in animal husbandry (OR, 3.9; 95% CI, 2.4 to 6.2), and raising and breeding pigs and pig farming, (OR, 6.6; 95% CI, 4.3 to 10.2) had a higher risk of acquiring *Blastocystis* infection, whereas those not working in agriculture (OR, 0.5; 95% CI, 0.3 to 0.8) and washing hands after defecation (OR, 0.5; 95% CI, 0.3 to 0.7) had a lower risk of the infection (Table 2). Multivariate analysis showed that participants residing in Non Sa Ard (Moo 11) or Tun Geang (Moo 18) Village were 1.9 times at higher risk (95% CI, 1.2 to 2.9) and those raising and breeding pigs as livestock were 5.4 times (95% CI, 3.4 to 8.5) at higher risk of acquiring *Blastocystis* infection than those who did not after adjusting all variable factors (Table 2).

**TABLE 2:** Univariate and multivariate analysis for risk factor of *Blastocystis* infection^*a*^

Variable factor	No. of enrolled subjects (%)	No. of infected subjects (%)	Raw values^*b*^		Adjusted values^*b*^	
			OR (95% CI)	*P* value	OR (95% CI)	*P* value
Sex						
Male	334 (44.8)	49 (14.7)	1			
Female	411 (55.2)	68 (16.5)	1.2 (0.8–1.7)	0.485		
Age (yr)	745 (100.0)	117 (15.7)	1.00 (0.99–1.01)	0.299	1.01 (0.99–1.02)	0.461
<20	108 (14.5)	11 (10.2)	1			
20–29	18 (2.4)	3 (16.7)	1.8 (0.4–7.1)	0.423		
30–39	66 (8.9)	6 (9.1)	0.9 (0.3–2.5)	0.814		
40–49	161 (21.6)	40 (24.8)	2.9 (1.4–6.0)	0.004		
50–59	202 (27.1)	32 (15.8)	1.7 (0.8–3.4)	0.173		
>60	190 (25.5)	25 (13.2)	1.3 (0.6–2.8)	0.450		
Living in Non Sa Ard or Tun Geang Village						
No	569 (76.4)	71 (12.5)	1			
Yes	176 (23.6)	46 (26.1)	2.5 (1.6–3.8)	<0.01	1.9 (1.2–2.9)	0.008
Occupation						
Agriculture	426 (57.2)	61 (14.3)	1			
Animal career	102 (13.7)	40 (39.2)	3.9 (2.4–6.2)	<0.01		
Other	217 (29.1)	16 (7.4)	0.5 (0.3–0.8)	0.012		
Education						
Primary school or below	559 (75.0)	97 (17.4)	1			
Middle school or above	186 (25.0)	20 (10.8)	0.6 (0.3–1.0)	0.034	0.7 (0.4–1.3)	0.250
Washing hands before eating						
No	206 (27.7)	36 (17.5)	1			
Yes	539 (72.3)	81 (15.0)	0.8 (0.5–1.3)	0.412		
Washing hands after defecation						
No	278 (37.3)	62 (22.3)	1			
Yes	467 (62.7)	55 (11.8)	0.5 (0.3–0.7)	<0.01	0.7 (0.4–1.1)	0.092
Raising and breeding chickens						
No	414 (55.6)	63 (15.2)	1			
Yes	331 (44.4)	54 (16.3)	1.1 (0.7–1.6)	0.683		
Raising and breeding cows						
No	539 (72.3)	81 (15.0)	1			
Yes	206 (27.7)	36 (17.5)	1.2 (0.8–1.8)	0.412		
Raising and breeding pigs						
No	606 (81.3)	59 (9.7)	1			
Yes	139 (18.7)	58 (41.7)	6.6 (4.3–10.2)	<0.001	5.4 (3.4–8.5)	<0.001

a Data were adjusted for sex, age, living village, occupation, education, washing hands before eating, washing hands after defecation, and raising and breeding livestock, i.e., chicken, cow, and pig.

b OR, odds ratio; CI, confidence interval.

### Characterization and genetic distribution of *Blastocystis* subtypes among human and pig.

**FIG 1:**
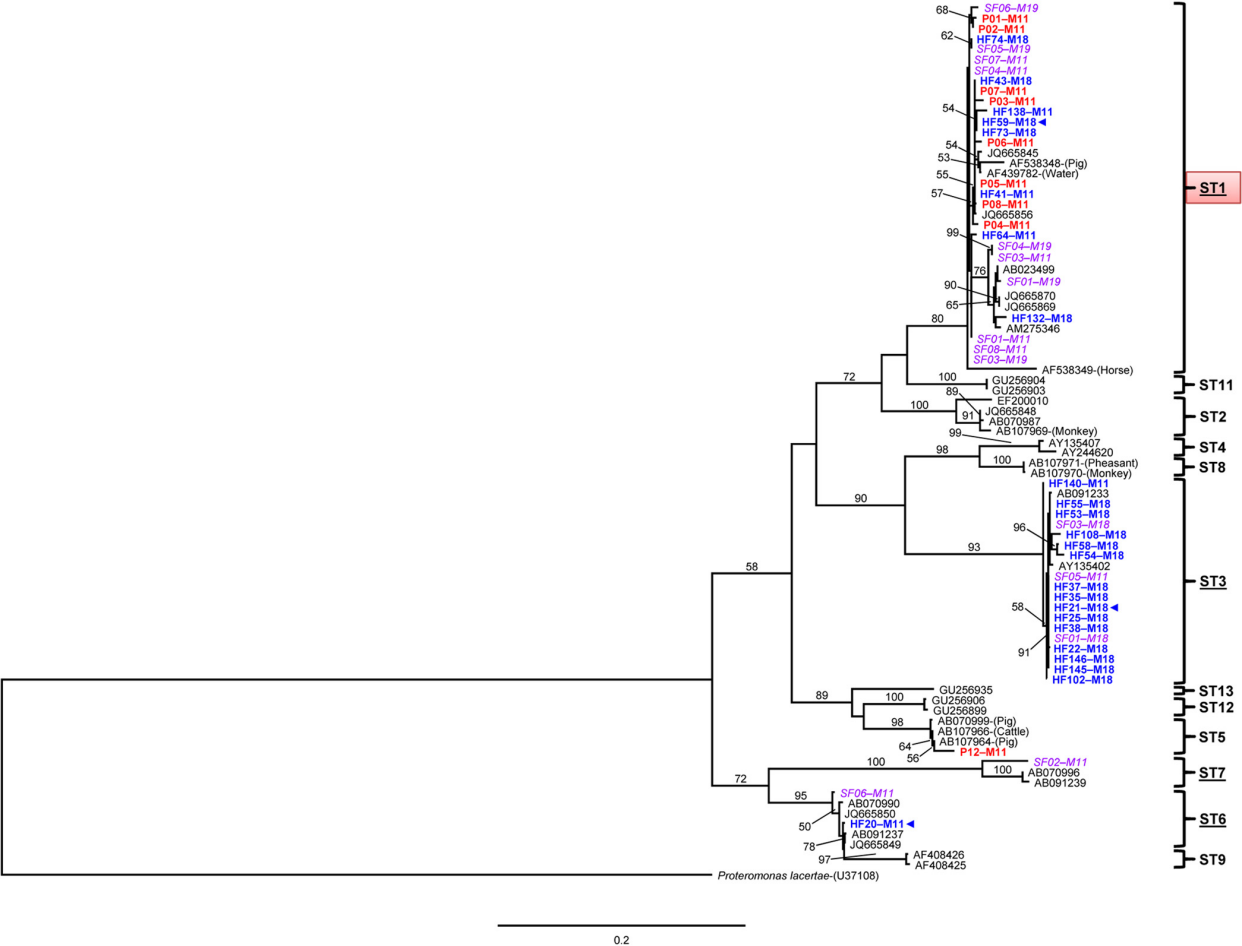
Randomized axelerated maximum likelihood (RAxML) tree of *Blastocystis* sp. based on small subunit rRNA (SSU rRNA) gene sequences. *P. lacertae* was chosen as an outgroup. Bootstrap values (1,000 replicates) are given as percentages above the individual branches. Branches with values of <50% are not shown. *Blastocystis* sp. isolated from schoolchildren’s feces (SF) are indicated in purple italics, participants’ feces (HF) in blue boldface, and pig feces (P) in red boldface, where the subtypes (STs) of humans are underlined and resident community number or Moo (M) is assigned after each fecal sample name. Animal isolates were labeled in parentheses after the accession number. Blue ◂, indicate *Blastocystis* isolated from open pig farm owners. The red dialog box indicates a major subtype with broad distribution across different communities.

Of 117 cultured positive human stool samples, PCR amplification was successful for 95 samples (81.2%). Small subunit (SSU) rRNA amplicons of 24 samples associated with residing in a village (Non Sa Ard or Tun Geang Village in Moo 11 and 18, respectively) and pig livestock were chosen randomly for sequencing. In addition, nine positive pig feces samples from an open pig farm of infected owners were amplified successfully and sequenced. In total, 33 sequences from humans (*n *= 24) and pigs (*n *= 9) showed a 98% to 99% genetic identity match with the SSU rRNA gene of *Blastocystis* species based on BLASTN. A phylogenetic tree of *Blastocystis* sp. based on 1,032 bp of the SSU rRNA gene was constructed using 33 DNA sequences in this study and 34 *Blastocystis* references retrieved from GenBank (Fig. 1). Proteromonas lacertae was used as an outgroup. The rooted randomized axelerated maximum likelihood (RAxML) tree identified 12 clades called ST1, ST2, ST3, ST4, ST5, ST6, ST7, ST8, ST9, ST11, ST12, and ST13 that were strongly supported by 80% to 100% bootstrap values. The tree topology revealed that *Blastocystis* subtypes observed among human samples in these two communities could be classified in four subtypes, i.e., ST1, ST3, ST6, and ST7, and formed a polyphyletic evolution. Subtypes 1 and 3 were predominant among people (*n *= 39; in which 24 sequences in this study and 15 sequences from Ruang-areerate et al. [25]) and found in these communities (46.2%, *n *= 18; 46.2%, *n *= 18, respectively), whereas other subtypes were comparatively less common, namely, subtypes 6 (5.1%, *n *= 2) and 7 (2.5%, *n *= 1). Interestingly, *Blastocystis* isolates of pig feces were found to be associated mostly with human subtypes (ST1, *n *= 8), otherwise, 1 isolate of *Blastocystis* from pig feces was clustered in the animal subtype (ST5, *n *= 1). Three owners of the open pig farm were infected with *Blastocystis* belonging to ST1, ST3, and ST6.

**FIG 2:**
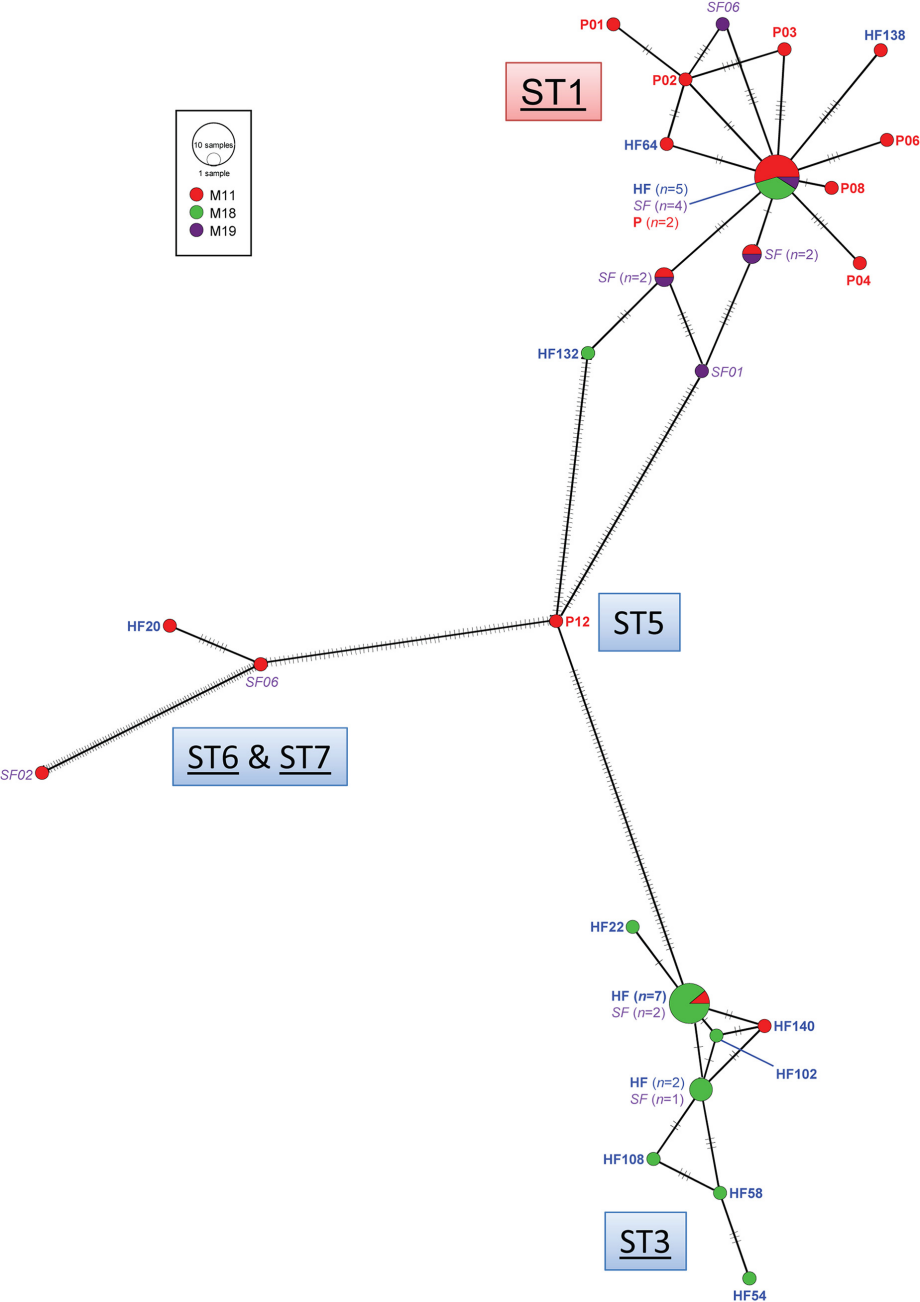
Minimum spanning network inferred from SSU rRNA gene sequences of *Blastocystis* sp. in the communities (Moo 11, red; 18, green; 19, purple) at Thakradan Subdistrict, Chachoengsao Province, eastern Thailand. Each singleton is labeled using a sample code. Common haplotypes are presented in size with the number of *Blastocystis* isolates. *Blastocystis* sp. isolated from schoolchildren’s feces (SF) are indicated in purple italics, participants’ feces (HF) in blue boldface, and pig feces (P) in red boldface, where the subtypes (STs) of humans are underlined. The red dialog box indicates a major subtype with broad distribution across different communities.

The haplotype diversity (Hd) and nucleotide diversity (Pi, π) among 48 *Blastocystis* isolates based on the SSU rRNA gene was 0.915 ± 0.028 and 0.056 ± 0.004, respectively (Table 3). The high Hd indicated that the *Blastocystis* population had high probabilities of a recombination rate leading to a large number of closely related haplotypes due to high genetic diversity. However, low nucleotide diversity (π) implied that small differences between haplotypes were found within a population. In all, 26 haplotypes of *Blastocystis* infection were found among people in these communities where the highest number of haplotypes was found within the population of Moo 11 (*n *= 22; Hd, 0.935 ± 0.047; π, 0.044 ± 0.011). In addition, the haplotype network clearly demonstrated two distinct groups of haplotypes related with ST1 and ST3 (Fig. 2). The common haplotype of ST1 was shared between human and two pig isolates of *Blastocystis* with closely related singletons of humans and pigs around it. The overall Tajima’s *D* and Fu’s *Fs* tests were positive (*D *= 0.08, *Fu *= 5.75, respectively) indicating an excess of high- and low-frequency polymorphisms causing population contraction or balancing selection (Table 3). Nonetheless, the *P* value accepted the null hypothesis suggesting a neutral population.

**TABLE 3:** Genetic diversity and neutrality test of the population of *Blastocystis* sp. in the communities at Thakradan Subdistrict, Chachoengsao Province, eastern Thailand using SSU rRNA gene sequences

Population	Sample size (*n*)	Haplotype	No. of polymorphic sites	Genetic diversity^*a*^		Result for neutrality tests^*b*^	
				Hd ± SD	Pi (π) ± SD	Tajima’s *D*	Fu’s *Fu*
Moo 11	22	17	162	0.935 ± 0.047	0.044 ± 0.011	–1.23	1.13
Moo 18	21	9	95	0.824 ± 0.066	0.037 ± 0.009	1.09	10.62
Moo 19	5	5	11	1.000 ± 0.126	0.006 ± 0.001	–0.58	–1.25
Total	48	26	169	0.915 ± 0.028	0.056 ± 0.004	0.08	5.75

a Hd, haplotype diversity; Pi (π), nucleotide diversity.

b No values were statistically significant, at a *P* value of >0.05.

## DISCUSSION

This study showed that the prevalence of *Blastocystis* infection was apparently high compared with that of related community studies (15.7% versus 6.1% to 7.8%). The Phra Ploeng community was located next to these communities (28, 29), suggesting that the *Blastocystis* infection remained persistent and distributed mainly in this eastern area. The prevalence and incidence might have been underestimated due to single stool collection and examination; nonetheless, the *in vitro* culture was found to be a highly sensitive and cost-effective screening method to estimate the prevalence of *Blastocystis* infection (30, 31). In 2015, the survey of *Blastocystis* infection among schoolchildren from four primary schools located in our studied communities demonstrated a prevalence of 12.9%; however, after implementing water treatment programs, the overall reduction of the infection was 4.8% in 2016 (25). Although spontaneous recovery is quite common among people living in this region (28), the *Blastocystis* infection among people in the community remains a problem probably due to reinfection by a persistent transmission route, e.g., untreated water (23, 25) or animal contact (22, 28, 32, 33). Interestingly, raising and breeding pigs in an open farm was shown to be one important risk factor that put people at a 5.4 times at higher risk of acquiring *Blastocystis* infection than those who did not reside in these communities. Many studies have shown evidence of different routes of *Blastocystis* transmission, such as person-to-person, waterborne, and zoonotic transmission, depending on the study population (23, 24, 34–38). Although the waterborne route was shown to be an important control mode of transmission in four primary schools of this community (25), this study demonstrated evidence of a zoonotic route of *Blastocystis* transmission in that humans and pigs shared identical *Blastocystis* in ST1.

Based on the SSU rRNA gene, *Blastocystis* sp. can be classified in at least 17 subtypes (13, 17, 38–40). The RAxML tree showed a tree topology similar to that in a related study in the same communities (25) in that 12 subtypes have been classified with highly supported bootstrap values (≥80%) in all node divergences. In these two communities, the *Blastocystis* infection among humans and pigs was polyphyletic and could be classified in five subtypes (ST1, ST3, ST5, ST6, and ST7), similar to related findings (25); however, ST5, a livestock subtype, was additionally found in one of nine pigs in raising and breeding operations in an open pig farm located in Moo 11. Otherwise, eight of all *Blastocystis* samples isolated from pig feces belonged to ST1, the predominant human subtype in the communities. The haplotype network has obviously demonstrated two distinct groups of haplotypes related to ST1 and ST3. Two of nine *Blastocystis* isolates from pig feces shared the same common haplotype as humans in ST1. A large number of closely related haplotypes was observed in this area, probably due to high probabilities of the recombination rate within populations of *Blastocystis* because of close contact among local people between communities. Moo 11 was shown to maintain the highest number of haplotypes. Tajima’s *D* and Fu’s *Fs* tests indicated that *Blastocystis* populations within these communities were under randomly evolving mutations or neutral populations; however, further study is needed owing to the limited population size in each community (41). Many studies have reported a similar concordance that subtype 1 is frequently observed and predominant in Thailand (23, 39) denoting that *Blastocystis* subtypes had a wide diversity and were generally distributed within the communities. Despite zoonotic and human infection, ST1 and ST3 constituted the majority of subtypes. Regarding the haplotype network, the distribution of ST1 was found commonly in three communities, including Moo 11, 18, and 19, whereas infection with ST3 strictly occurred in Moo 18 suggesting an unequal distribution of *Blastocystis* populations in this region. Environmental factors associated with the distribution of different *Blastocystis* subtypes remain unknown, and there is a need to understand and eliminate all potential risk factors that could cause reinfection among people in the communities. Hence, the evidence of zoonotic transmission of *Blastocystis* infection has demonstrated that *Blastocystis* isolates in ST1 were closely identical and some shared the same common haplotype between breeding pigs (*n *= 8) and open pig farm owners (*n *= 1) as well as people living in same (Moo 11) and different communities (Moo 18 and 19). Thus, the zoonotic subtype 1 could cause an infection across these three communities of the Thakradan subdistrict where pig feces by-products from open farm systems are commonly used for producing organic fertilizer. The pig feces fertilizer is distributed normally for plant breeding and cultivation within the communities owing to ecotourism and sufficiency economy theory. Nevertheless, isolation and characterization of *Blastocystis* in the fertilizer are further needed to confirm the existence of fertile cysts that could be the causative sources of reinfection and distribution of ST1 in these communities.

ST1 has been reported both in humans and a broad range of animals, i.e., pigs, horses, monkeys, cattle, rodents, chickens, quail, and pheasants, implying a potential zoonotic transmission (32, 40, 42). Noël et al. (42) demonstrated that subtype 1 was not host specific and cross-infective among various animals. Questions have been raised about the source of the *Blastocystis* contaminant related to the modes of transmission of *Blastocystis* sp. According to the large number of potential zoonotic hosts, host origins and transmission routes remain difficult to identify and control, unlike the control of waterborne transmission by untreated water and poor sanitary conditions (25). This finding provides important evidence demonstrating the potential source of the *Blastocystis* contaminant in the zoonotic route among people in this population. Currently, organic fertilizer is considered better than chemical fertilizer in the long-term effects on land after cultivation and plantation due to being cost-effective and harmless environmentally. Thus, properly treated organic fertilizer from natural animal feces by-products with standard production as well as protective contact could be one key solution to control and prevent zoonotic transmission of *Blastocystis* infection, particularly in agricultural communities. Even though Jones’s media are suitable for field work study of *Blastocystis* infection which high yield of DNA could be obtained for subtype identification, unfortunately, the dominant subtype in the culture might have affected the detection of other subtypes by having more DNA during the isolation step, which was one of the limitations in this study. Direct DNA stool extraction that has high sensitivity is suitable for detecting actual host colonization and would increase the prevalence of *Blastocystis* infection; however, molecular diagnostic testing was supported insufficiently at the lab station due to limitations, such as sophisticated equipment and a sterile working area.

In conclusion, we have demonstrated a potential source of zoonotic transmission of *Blastocystis* sp. in persistently reinfected communities of eastern Thailand where mixed infection and nonrestricted distribution were common. ST1 remained one major cause of the infection and was associated with the zoonotic risk factor pig feces; however, the source of transmission of the other major ST3 and minor ST5, ST6, and ST7 remains unclear. The closely identical genetic background of *Blastocystis* sp. in ST1 between humans and animals and pigs raised and bred in open farms highly suggested evidence of potential zoonotic transmission. Regarding the distribution of pig feces by-products for the use of organic fertilizer in the communities, the contact during agriculture for plant cultivation could be one important key factor causing the reinfection of ST1 among people in the communities. Hence, this evidence provokes attention to properly treat organic fertilizer from animal feces by-products using standard production and protective contact to sustainably reduce the morbidity of intestinal *Blastocystis* infection among people in agricultural communities, especially where the organic fertilizer depends on animal feces by-products.

## MATERIALS AND METHODS

### Study population and specimen collection.

A cross-sectional study of *Blastocystis* infection was conducted in 11 villages of 2 communities (6 villages in Moo 11 and 5 villages in Moo 18) at Thakradan Subdistrict, Chachoengsao Province, eastern Thailand in January 2018, 153 km from Bangkok and 50 km from the Cambodian border. The research protocol was reviewed and approved by the Ethics Committee of the Royal Thai Army Medical Department (reference number S025q/61_Exp). Informed consent was obtained from the enrolled participants (*n *= 902). Demographic information was collected, including associated risk factors (10, 14–16, 21–24) and clinical symptoms of *Blastocystis* infection. A face-to-face interview was conducted for every household visit using electronic standardized questionnaires. Each participant received a prelabeled container and was advised regarding the method of a single stool collection. Stool specimens (*n *= 745) were examined for intestinal parasitic infections under a light microscope by wet preparation, the Kato-Katz technique, and formalin ethyl acetate concentration at the microscopic lab station in the field.

In addition to human feces, pig feces (*n *= 12) were collected from two local pig farms at Moo 11 associated with a *Blastocystis* risk factor. Each fecal sample was collected carefully from the upper portion of fresh defecated material in order to avoid ground contamination, which is the same as the stool collection technique in humans; then, samples were kept individually in a sterile container. Before collecting fecal samples, the pigs’ owners were informed verbally about the project and the collecting protocol. All owners provided their verbal informed consent personally and permission to collect feces from their pigs. The pigs were raised freely and bred under an open system, and their feces by-products were further used to produce organic fertilizer for organic sufficiency agriculture and agricultural heritage/agrotourism in the community. Fecal samples were examined in a manner similar to the stool examination of the human participants as described previously.

### DNA extraction and PCR amplification.

Short-term *in vitro* cultivation of *Blastocystis* sp. was performed using Jones’ medium supplemented with 10% horse serum (30, 43, 44). Samples were incubated at 37°C for 2 to 3 days and examined for *Blastocystis* sp. by light microscopy. Infected cases were defined as the detection of vacuolar, multivacuolar, or other forms of *Blastocystis* sp. in the *in vitro* culture of a stool specimen. The positive samples from culture medium (*n *= 117) were centrifuged at 2,300 × *g* for 10 min to precipitate *Blastocystis* pellets before extracting DNA. The pellet samples were extracted using the DNeasy blood and tissue kit (Qiagen, Hilden, Germany) as described by the manufacturer instructions. The extracted DNA was eluted to a 100-μl final volumes and stored at −20°C until used. PCR amplicons of the small subunit rRNA (SSU rRNA) gene of *Blastocystis* that were approximately 1,790 bp were amplified using the MJ Mini thermal cycler (Bio-Rad, Hercules, CA, USA) according to Yoshikawa et al. (34). Electrophoresis was performed with the PCR products on 1% agarose gel, and the gel was visualized using a Molecular Imager Gel Doc XR+ system with Imager Lab 3.0 software (Bio-Rad).

### DNA sequencing and phylogenetic analysis.

To subtype and characterize the genetic relationships of *Blastocystis* infection between humans and pig livestock in the communities, 33 positive PCR products, of which 24 were from humans and 9 were from pigs, were purified and sent to Bionics Co. Ltd. (Seoul, South Korea) for direct sequencing. The sequencing chromatograms were validated and edited manually using BioEdit version 7.2.5 (45). Nucleotide sequences were identified for *Blastocystis* infection using NCBI BLAST search (https://blast.ncbi.nlm.nih.gov/Blast.cgi) (46). To generate the phylogenetic tree, the SSU rRNA gene sequences of *Blastocystis* obtained in this study were multiple aligned with a set of 34 *Blastocystis* sequences and a *Proteromonas lacertae* sequence as an outgroup (42), which was retrieved from GenBank database using ClustalW in BioEdit version 7.2.5. The randomized axelerated maximum likelihood (RAxML) tree was constructed based on RAxML version 7.4.2 with a GTR matrix (GTR + Γ model) (47) using RaxmlGUI version 1.3 (48) where clade stability was evaluated using 1,000 replicates of RAxML bootstrap values.

### Population genetic analysis.

To determine the genetic diversity of the SSU rRNA gene among 48 *Blastocystis* isolates (33 in this study and 15 from a previous study conducted by Ruang-areerate et al. [25] at this community), haplotype diversity (Hd) and nucleotide diversity (Pi) were calculated using DnaSP version 6.0 (49). To investigate relationships among haplotypes, the haplotype network was constructed using a minimum spanning haplotype network with POPART version 1.7 (50). A selective neutrality test was used to determine genetic hitchhiking, population expansion, selective sweep ,and bottleneck using the statistical significance of Tajima’s *D* and Fu’s *Fs* tests at a 95% interval (*P* < 0.05) (51, 52).

### Statistical analysis.

The association between potential risk factors and *Blastocystis* infection was analyzed using STATA/SE version 14 (StataCorp LP, College Station, TX, USA). The prevalence was reported with percentage, and risks were assessed with odds ratio (OR) with 95% confidence interval (CI) and *P* value. A univariate analysis was performed initially to determine relationships between the study covariate and infection status (yes/no). Logistic regression was performed for the multivariate analysis to assess the independent association of risk factors and *Blastocystis* infection.

### Data availability.

All sequences in this study were submitted to GenBank under accession no. MZ164983 to MZ165006 and MZ165024 to MZ165032.
